# Wider Retinal Artery Trajectories in Eyes with Macular Hole Than in Fellow Eyes of Patients with Unilateral Idiopathic Macular Hole

**DOI:** 10.1371/journal.pone.0122876

**Published:** 2015-04-13

**Authors:** Naoya Yoshihara, Taiji Sakamoto, Takehiro Yamashita, Toshifumi Yamashita, Keita Yamakiri, Shozo Sonoda, Tatsuro Ishibashi

**Affiliations:** 1 Department of Ophthalmology, Kagoshima University Graduate School of Medical and Dental Sciences, Kagoshima, Japan; 2 Department of Ophthalmology, Kyushu University, Fukuoka, Japan; Saitama Medical University, JAPAN

## Abstract

**Purpose:**

To determine whether the width of the retinal artery (RA) trajectory was associated with the presence of a macular hole (MH).

**Methods:**

A retrospective cross sectional case-control study was performed. The fundus photographs were rotated 90 degrees, and the coordinates of the best fit curve of the RA trajectory were determined automatically based on these plots using the ImageJ program. The converted coordinates were fit to a second degree polynomial (ax^2^/100 + bx + c) equation. The width and steepness of the RA trajectory, “a”, of the eyes with a MH eye were compared to that of the fellow eyes.

**Results:**

One hundred and ten eyes of 55 consecutive patients (30 women) with a unilateral MH and healthy fellow eyes were analyzed. The mean age was 64.9 years (range 47-81 years). The constant ‘a’ was significantly smaller in eyes with a MH than that of the fellow eyes (0.379 ± 0.094 vs 0.416 ± 0.121, *P* = 0.001, paired *t* test), indicating that the RA trajectory was wider in the MH eyes than in the fellow eyes. There was a significant correlation between the axial length and ‘a’ of the RA trajectory in the MH eyes (R = 0.273, *P* = 0.044) and in the fellow eyes (R = 0.356, *P* = 0.008; Spearman’s rank correlation coefficient).

**Conclusions:**

Because eyes with a MH have a significantly wider and flatter RA trajectory, there may be greater traction on the fovea which is located between the RA arches. The causative role of this finding is still unclear.

## Introduction

The pathologic mechanism causing the development of an idiopathic macular hole (MH) was first stated by Gass [1,2]. His concept was that an anterior traction on the fovea is generated by the posterior hyaloid membrane in eyes with a posterior vitreous detachment (PVD) [3–8]. This concept of traction as the cause of MH development has been supported by optical coherence tomography (OCT), and it is now widely accepted that traction at the level of the vitreofoveal interface is the underlying mechanism for idiopathic MH formation.

However, there are still several unanswered questions. For example, what causes the traction by the posterior hyaloid on the retinal surface, and how does the fragility and stiffness of the retina affect the formation of a MH. Because a MH has a higher incidence in eyes with longer axial lengths (myopia) and in older individuals, especially women, we can reasonably assume that the structure of the eye and changes in the structure with increasing age could be involved in the pathogenesis of MHs [9–11]. For example, it has been shown that the retinal thickness decreases with increasing age which should make the retinas of older individuals more susceptible to MH formation [11]. In addition, the incidence of MHs is higher in highly myopic eyes than in emmetropic eyes [9]. This is most likely because myopic eyes have longer axial lengths and thinner retinas.

The adhesion of the vitreous to the retinal arteries is relatively strong [12], and this must be considered in the pathogenesis of MHs. If two points are connected by elastic tissues such as a posterior vitreous membrane, the moment of inertia or momentary force generated by ocular movements is determined by the weight of the tissues between the two points [13]. Thus, the momentary tractional force on the macula might be greater in eyes with a wider RA trajectory as it is in eyes with a MH.

These structural changes will affect the biophysical properties of the retina. Thus, it seemed reasonable to examine the structure of eyes with a MH in more detail. Because the development of a MH cannot be predicted, examination of an eye prior to the development of a MH is not possible. Thus, experiments to answer these questions have not been performed.

The results of earlier studies showed that there are two peaks in the retinal nerve fiber layer (RNFL) thickness profile in normal eyes; the supratemporal peak and the infratemporal peak [14–19]. Because the fovea is located between these two peaks, the retina will be stretched more horizontally and/or vertically in eyes with a greater distance between these two peaks. This may result in not only a thinner retina but also an increase in the tension on the fovea.

We recently reported that the location of the peaks of the RNFL thickness were significantly correlated with the location of the major retinal arteries (RAs) exiting the optic disc using a mathematical model [20]. Because a wider RA trajectory would exert greater traction on the macular area, we hypothesized that the trajectories of the RAs will be wider in eyes with a MH than in the fellow eyes. To test this hypothesis, we compared the RA trajectories of 55 eyes with a MH to that in the fellow eyes. To the best of our knowledge, there has not been a study comparing the RA trajectories in eyes with a MH.

## Methods

This was a retrospective, cross sectional, case-control study carried out with the approval of the Institutional Review Board of Kagoshima University Hospital, Kagoshima, Japan. The procedures used adhered to the tenets of the Declaration of Helsinki. The Institutional Review Board also approved the retrospective collection of the data from the medical charts of the patients with and without retinal diseases. Prior to the MH surgery, a written informed consent was obtained not only for the surgery but also for the use of the data for future research studies.

Among the 71 consecutive cases with a unilateral MH examined between November 2011 to May 2014, 16 cases were excluded; 2 cases had unilateral retinal vein occlusion, 3 cases had retinal degeneration, 1 case had abnormal vascular tortuosity, and 10 cases had insufficient data due to poor fundus photographs. In the end, 55 cases with healthy fellow eyes were studied. None of the studied cases had any signs of other retinal diseases including diabetic retinopathy, retinal detachment, and glaucoma.

### Ophthalmic examinations including measurements of axial lengths and refractive errors

All of the eyes had a standard ocular examination including slit-lamp biomicroscopy of the anterior segment, ophthalmoscopy of the ocular fundus, IOP measurements with a pneumotonometer (CT-80, Topcon, Tokyo, Japan), and axial length measurements with the AL-2000 ultrasound instrument (Tomey, Japan). The procedures for these examinations were explained in detail in our earlier publications [17,20,21]. In addition, the refractive errors (spherical equivalent) were measured with the Topcon KR8800 auto-refractometer/keratometer.

### MH staging and diagnosis of posterior vitreous detachment (PVD)

Optical coherence tomography (OCT) with the 3D OCT-1000 Mark II (Topcon) was used to determine the stage of the MH as in our earlier studies [2,22]. The presence of a PVD was determined by the OCT images and echography. The diagnosis was made by two masked observers (NY, KY), but when the diagnosis was split, a third grader (TS) made the final decision.

### Calculation of retinal artery (RA) trajectories

**Fig 1:**
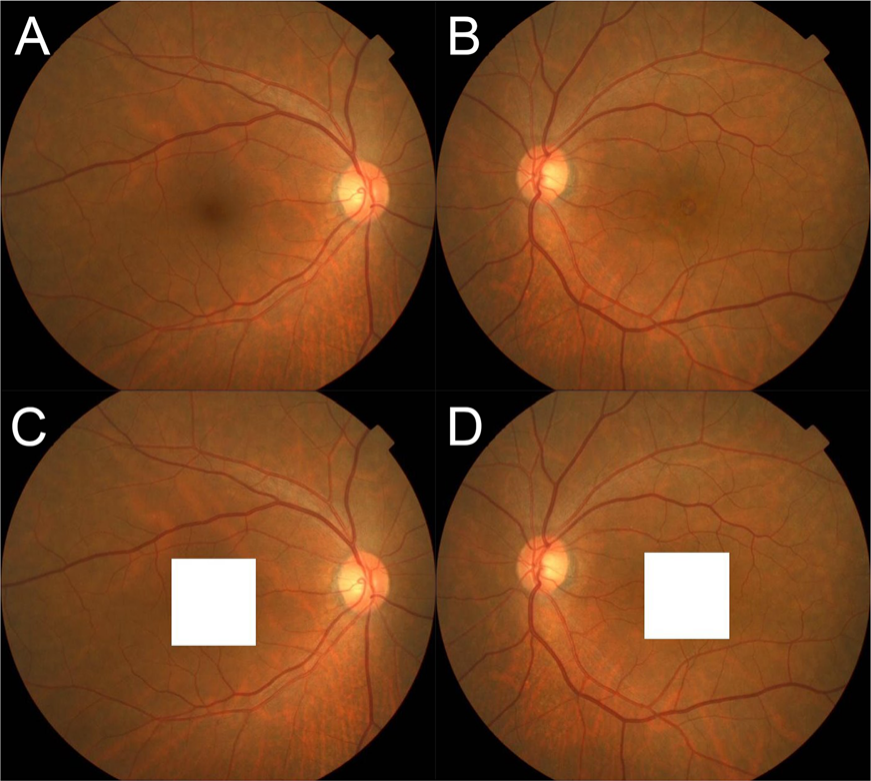
Fundus photographs of eye with macular hole and fellow eye. This case had macular hole in left eye (A and B). To mask the laterality of diseased eye, macular area of both eyes were covered by the square shield (C and D).

The calculation of the RA trajectories was done with a mathematical model as described in detail [20]. To do this, standard fundus photographs were taken of each eye with a digital fundus camera equipped with OCT (Topcon 3D OCT-1000 Mark II, Topcon). To mask the MH eyes from the examiner, the macular area of the fundus image was covered with square shield prior to the analyses (Fig 1).

**Fig 2:**
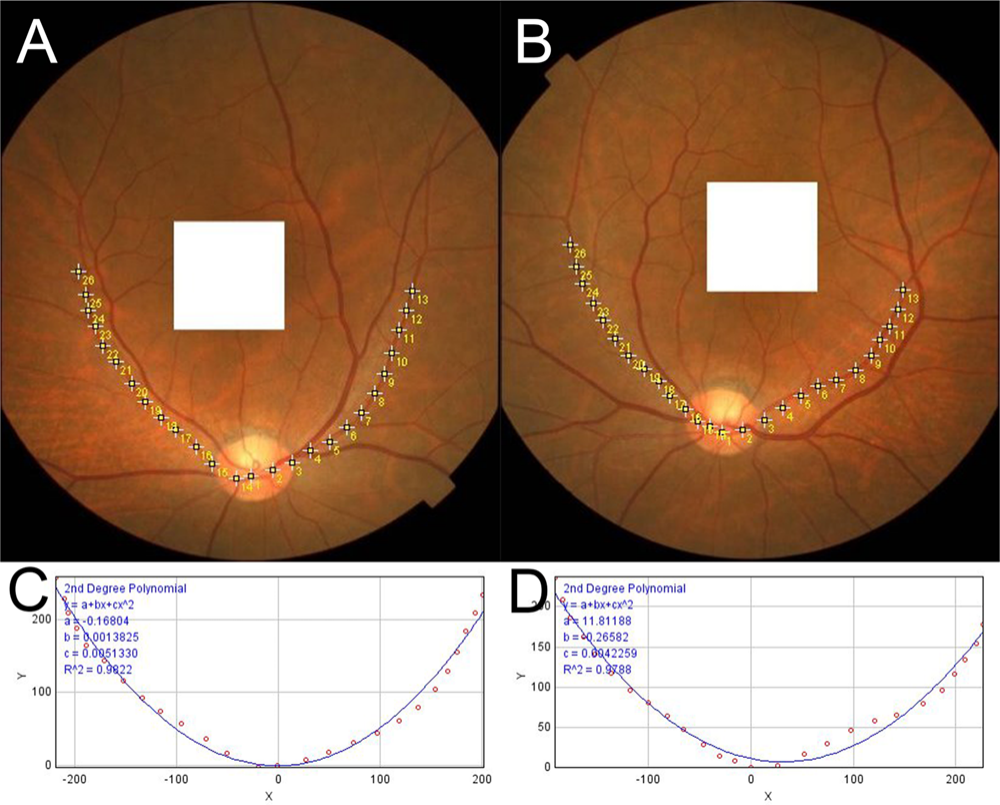
Trajectories of retinal arteries. The fundus photograph was rotated 90 degrees clockwise for the right eye and 90 degrees counterclockwise for the left eye (A and B). The retinal arteries were marked by at least 20 points. The coordinates of the marked spots were determined automatically using ImageJ software and converted to a new set of data with the center of the optic disc as the origin. The converted coordinate data were fit to a user-defined second degree polynomial equation (ax^2^/100 + bx + c) (C and D).

Next, the fundus images were rotated 90 degrees clockwise for the right eyes and 90 degrees counterclockwise for the left eyes. Then, 20 or more loci were selected that were located on the RA trajectories, and the loci were marked on the fundus photographs (Fig 2).

The ‘x’ and ‘y’ coordinates of each locus were determined automatically with the ImageJ program (ImageJ version 1.47, National Institutes of Health, Bethesda, MD; available at: http://imagej.nih.gov/ij/). The coordinates were then converted to a new set of ‘x’ and ‘y’ coordinates with the center of the disc as the origin. Finally, the converted coordinate data were fit to a second degree polynomial equation, ax^2^/100 + bx + c, with the curve fitting program of ImageJ. The ‘a’, ‘b’, and ‘c’ are constants calculated by the curve fitting program of ImageJ. The ImageJ curve fitter program determined the best fit second degree polynomial equation by the least squares method automatically. Under these conditions, a larger ‘a’ will make the curve steeper and narrower and the arms of the curve will be closer to the fovea. Thus, the ‘a’ constant was used to designate the width and steepness of the RA trajectory.

In eyes in which the RA branched, the plotting was made with the following rules. If a branch artery was smaller than the main artery, the main artery was used to determine the RA trajectory. If the branch artery was as large as the main artery, the plotting was not done after the branching point. Even so, the plotting was done for more than 20 points as described in our earlier publication [20].

### Retinal artery (RA) trajectory and posterior vitreous detachment in fellow eyes

The correlation of ‘a’ of the RA trajectory and the status of posterior vitreous detachment was analyzed for the fellow eyes. The status of the posterior vitreous detachment was classified into a previous classification [23], where S0 represents no posterior vitreous detachment and S4 represents a complete detachment. The significance of the correlations was determined by Mann-Whitney U test.

### Correlation with other factors

The correlations of RA trajectories and the size of the MH, stage of the MH, visual acuity (logarithm of the minimal angle of resolution, logMAR units), and duration of symptom were also determined. The duration of the symptoms was determined from the course of the disease process.

### Statistical analyses

All statistical analyses were performed with the statistical programming language R (version 3.0.2, The R Foundation for Statistical Computing, Vienna, Austria). The correlation between the RA trajectory and the axial length, the size of the MH, visual acuity, and duration of the symptoms was determined by Spearman’s ranked correlation analyses. The correlation between the RA trajectory and stage of MH was determined by Steel-Dwass tests. The constant ‘a’ of the RA trajectory in MH eyes was compared to that of the fellow eyes. Paired *t* tests were used to determine whether differences in ‘a’ were significant. The differences of axial length and refractive errors were compared with Wilcoxon tests. A *P*-value of <0.05 was taken to be statistically significant.

## Results

The demographic data of the 110 eyes of the 55 cases (25 men and 30 women) are shown in Table 1. The mean age of the patients was 64.9 ± 7.2 years (± SD), and the mean refractive error was -0.45 ± 2.20 diopters (D) in eyes with a MH and -0.36 ± 2.36 D in the fellow eyes (*P* = 0.365, Wilcoxon test).

**Table 1:** Patient demographics.

	MH eyes	Fellow eyes	*P* value
Age (years)	64.9 ± 7.2 (47 ~ 81)		
Sex (M/F)	25 / 30		
Spherical equivalent (diopters)	-0.45 ± 2.20	-0.36 ± 2.36	0.365
	(-8.00 ~ 3.00)	(-8.50 ~ 3.00)	
Axial length (mm)	23.6 ± 1.0	23.5 ± 1.1	0.462
	(21.2 ~ 27.2)	(21.1 ~ 27.2)	
Retinal artery trajectory	0.379 ± 0.094	0.416 ± 0.121	0.001*
	(0.128 ~ 0.659)	(0.137 ~ 0.694)	

The values are expressed as mean ± standard deviation (Range);

MH = macular hole;

* P value < 0.05.

**Fig 3:**
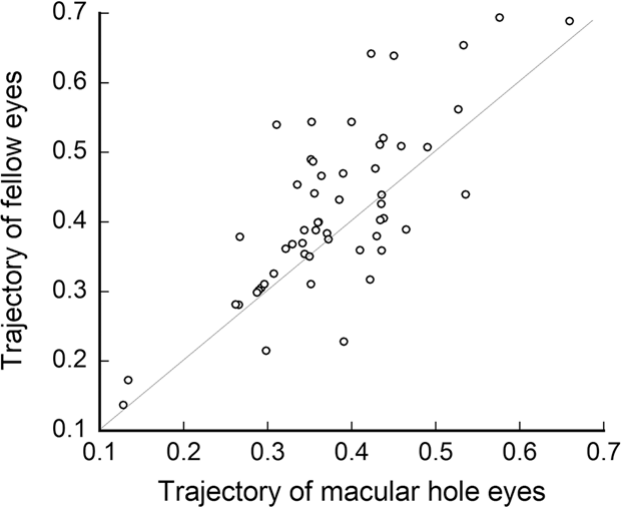
Scatter plot of eyes with macular hole (MH) and fellow eyes. The curve of retinal artery trajectory was expressed by a second degree polynomial equation, where a larger constant “a” made the curve steeper. Most of plots are distributed in the area above the diagonal line, indicating the “a” constant was significantly smaller in eyes with MH than fellow eyes (*P* = 0.001, paired *t* test).

**Fig 4:**
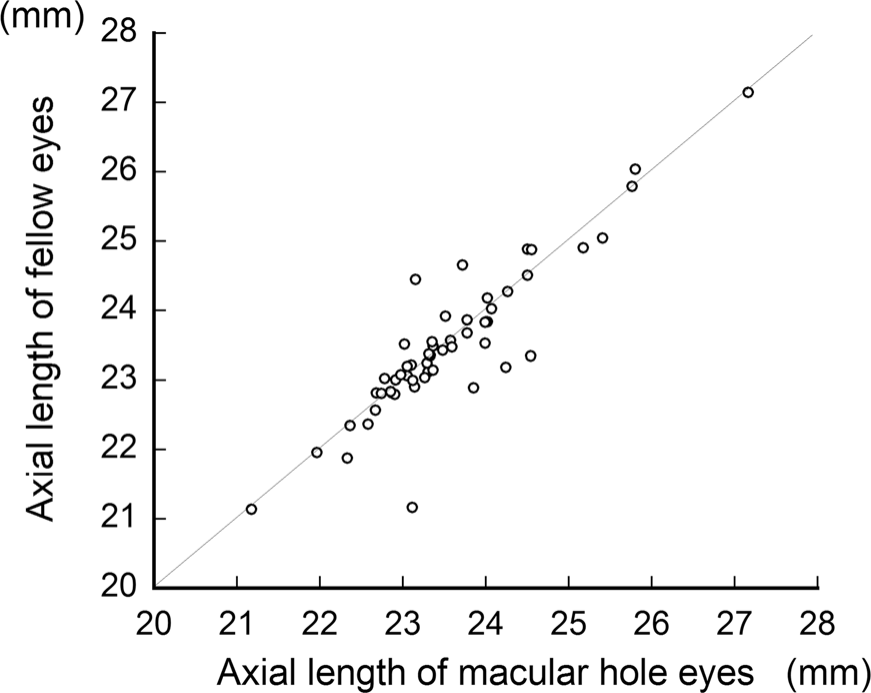
Axial length of macular hole eye and fellow eye (paired-eyes). There was no significant difference in the axial lengths between the MH eyes and fellow eyes (*P* = 0.462).

The curvature of the retinal artery trajectory as expressed by the constant ‘a’ was significantly flatter and wider in eyes with a MH (0.379 ± 0.094) than that in the fellow eyes (0.416 ± 0.121; *P* = 0.001, paired *t* test, Fig 3). The mean axial length in eyes with a MH was 23.6 ± 1.0 mm which was not significantly different from the 23.5 ± 1.1 mm in the fellow eyes (*P* = 0.462, Wilcoxon test, Fig 4).

### Correlation between ‘a’ of retinal artery (RA) trajectory and axial length

**Fig 5:**
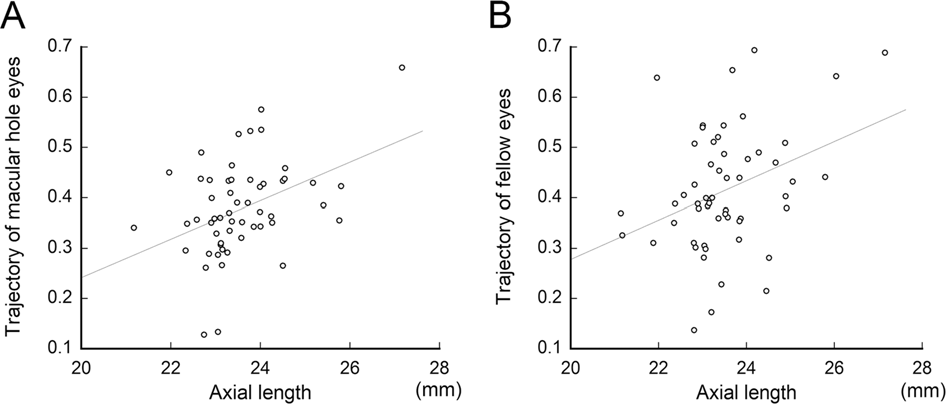
Scatterplots of the retinal artery trajectories and the axial lengths. The retinal artery trajectory, expressed as “a” was significantly and positively correlated with the axial length in eyes with a macular hole (A) and the fellow eyes (B).

In eyes with a MH, there was significant and positive correlation between the axial length and ‘a’ of the RA trajectory (R = 0.273, *P* = 0.044, Spearman’s rank correlation coefficient; Fig 5A). There was also a significant correlation between these two parameters/factors in the fellow eyes (R = 0.356, *P* = 0.008; Fig 5B). These results indicated that eyes with longer axial lengths had wider RA trajectories.

### Retinal artery (RA) trajectory and posterior vitreous attachment in fellow eyes

**Fig 6:**
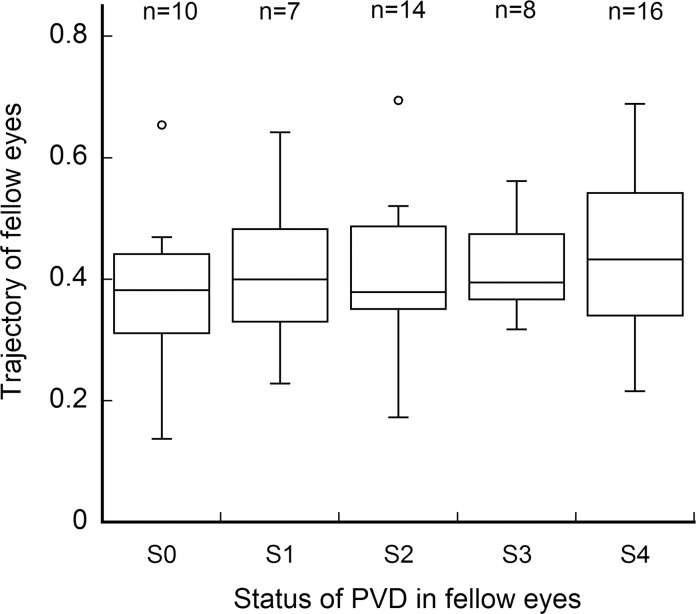
Retinal artery (RA) trajectory and posterior vitreous detachment in fellow eyes. The status of the posterior vitreous interface was classified according to Uchino et al [23]. There was no significant difference in the ‘a’ of the RA trajectory between any two of classifications including S0 and S4 (*P* = 0.370, Mann-Whitney U test).

In the 55 fellow eyes, 10 eyes were S0, 7 eyes were S1, 14 eyes were S2, 8 eyes were S3, and 16 eyes were S4. The average ± SD of the RA trajectory was: 0.389 ± 0.133 in S0, 0.414 ± 0.138 in S1, 0.407 ± 0.122 in S2, 0.419 ± 0.082 in S3, and 0.441 ± 0.133 in S4 (Fig 6). There was no significant difference between any two of posterior vitreous detachment status including S0 and S4 (*P* = 0.370, Mann-Whitney U test).

### Correlation between ‘a’ of the RA trajectory and other factors

The average size of the MH was 441.85 ± 186.75 micrometer (± SD), and no significant correlation was found between R trajectory and the size of MH (R = 0.112, *P* = 0.417, n = 55, Spearman’s ranked correlation analysis). Eight eyes were classified as stage 2, 35 eyes as stage 3, and 12 eyes as stage 4 MH. There were significant correlations between the ‘a’ of the RA trajectory and stage 2 and stage 3 eyes but not with stage 4 eyes (see supporting information S1 Fig).

The average visual acuity was 0.829 ± 0.362 logMAR units, and no significant difference was found between the ‘a’ of the RA trajectory and the visual acuity (R = 0.145, *P* = 0.29, n = 55, Spearman’s ranked correlation analyses).

The duration of the symptoms was determined in 44 eyes of 55 eyes, and the average duration of symptom was 2.9 ± 2.1 months. No significant difference was found between the ‘a’ of the RA trajectory and the duration of symptoms (R = 0.112, *P* = 0.417, n = 44, Spearman’s ranked correlation analyses).

Additionally, the ratios of the RA trajectory before and after surgery (RA trajectory before surgery/ RA trajectory after surgery) were compared. The results showed that the ratio was 1.00 ± 0.06 indicating that the RA trajectory was not significantly changed after vitrectomy (n = 37, *P* = 0.982, Wilcoxon test).

## Discussion

The results showed that the ‘a’ of the RA trajectory was significantly smaller in eyes with MH than in the fellow eyes. Therefore, the retinal arteries of eyes with a MH had wider parabola-shaped trajectory with flatter slopes than that of the fellow eyes resulting in a greater distance between retinal arteries and the fovea in eyes with a MH than in the fellow eyes. Our earlier study showed that the calculations of the RA trajectories using the mathematical model had high reproducibility and repeatability [20]. These results indicate that the present findings are valid.

The results can be interpreted in several ways. First, the findings indicate that the tractional forces of the posterior vitreous on the macula were probably greater in eyes with a MH because of the wider RA trajectory. In addition, it is known that a perifoveal PVD is more likely to develop under different conditions, e.g., increasing age, and this can generate an anterior tractional force on the macula. The increased traction can result in the formation of MHs [7,8,24]. As described in the Introduction section, if the distance between retinal arteries is large, it can generate stronger tractional forces on the fovea by inertia or momentary force during eye movements. Thus, the finding that eyes with a MH had smaller ‘a’ than that of their fellow eyes appears reasonable.

A second interpretation of the findings is that the structural characteristics and the fragility of the macula are related to the degree of RA trajectory. Our earlier study showed that the RA trajectory is significantly correlated with the RNFL thickness peak trajectory [20]. The macula is located between the RNFL thickness peaks, and the retina including macula would be stretched more in eyes with a wide RNFL peak than those with narrow widths during development and growth. It is possible that the stretched retina might make it thinner and more fragile. The fragility would then make the retina more susceptible to MH formation by the traction. Recently, Kumagai et al reported that the fovea of the fellow eyes of patients with a unilateral MH was significantly thinner and deeper than that of healthy eyes or eyes with other retinal diseases [25]. Considering that the fellow eyes of patients with a unilateral MH have a higher chance of developing a MH than healthy eyes [26–29], these eyes might represent eyes prior to the formation of a MH. Although we did not measure the foveal thickness, it might be possible that the stretched retina during development or after birth may become thinner than non-stretched retina. Thus, Kumagai et al’s findings are consistent with the present findings. However, these are correlations which support our hypothesis and are not related to causal relationships. It is necessary to measure the actual traction in future studies.

A third interpretation is that the RA trajectory became wider after the MH developed. However, in our earlier study of cases with bilateral MHs, fundus photographs were taken before and after MH formation in 3 eyes. We found that “a” changed from 0.340 to 0.341 in Case 1, from 0.146 to 0.150 in Case 2, and from 0.569 to 0.568 in Case 3 after the formation of the MH. Examination of the RA trajectories of these eyes showed that the RA trajectory was almost the same before and after MH formation; although three eyes were too small to conclude. Thus, this possibility that the wider RA trajectory developed after formation of MH is unlikely.

Although women and older individuals have a significantly greater risk of developing a MH, there are many other factors that can affect the development of a MH, such as axial length and the status of the posterior vitreoretinal interface [9–11,26–30]. The RA trajectory is also affected by these factors [20]. Thus, it would be difficult to collect suitable matched controls. According to our biostatistician, a paired-eye analysis was reasonable, and a practical approach to match the demographics of the cases and controls. It should be noted that MH eyes have a wider trajectory than fellow eyes, but not the healthy control eyes in this study. The comparative study between MH eyes and reasonable control eyes is planned in the near future.

It might be argued that the magnification effect may have affected the results obtained from fundus photographs [31]. The magnification effect is caused by different axial lengths. The concordance of the axial lengths of the MH and fellow eyes of patients with a unilateral MH is controversial [29]. In our cohorts, there was not a significant difference between the axial lengths of MH eyes and fellow eyes (Fig 4). Therefore, the magnification effect most likely did not affect the present results.

Another concern might be the influence of the rotation of the fundus photographs 90 degrees before the coordinates were determined. The fovea-optic disc line is not perfectly horizontal but slightly tilted. So it would be better if the rotation would be made so that the fovea-optic disc axis was vertical in all eyes. However, our earlier study showed that the ‘a’ constant was not affected by the 90 degree rotation compared with a case-by-case rotation [20]. Thus, the rotation of 90 degrees is a reasonable method.

This study has limitations. This was a cross sectional study, and it is not certain whether a MH will develop in some of the fellow eyes. In addition, the number of eyes was not large and thus a sampling bias cannot be excluded. The plotting of the RA trajectories was done in a masked fashion and the inter-rater agreement was very high in our earlier study [20]. However, the analyses were done by experienced examiners, and thus the methodology might not be as repeatable by other examiners.

We emphasize that we showed a wide RA trajectory is associated with the presence of a MH eyes, but do not state that a wide RA trajectory is the most important factor to cause the MH or a wide RA trajectory has a causative role in MH formation. Earlier studies showed that other factors can affect the development of a MH [9–11,25–29]. Rather, the present structural characteristics may affect or be affected by the factors such as a PVD, vitreoretinal adhesions, fragility of macular, axial length, and age. The status of PV interface is not uniform, e.g.,a perifoveal PVD or total PVD, however differences in the PV interface was not differentiated in this study. Spaide et al reported that the diameter of the vitreous attachment in eyes with perifoveal PVD was correlated with induced changes in foveal anatomy, and also that the stress may increase with smaller areas of attachment leading to mechanical failure of the macula [32]. This issue was not considered in our analyses. Additionally, the vertical OCT images including the arteries and the macula using the current wide-viewing OCT machine may be more helpful for studying the effect of the RA trajectory and vitreoretinal traction. It is necessary to examine a larger number of eyes with well-matched demographics and follow them longitudinally for a better conclusion. These limitations should be remembered when interpreting the results.

In conclusion, our results show that the RA trajectory was wider and flatter in MH eyes than fellow eyes. These retinal structural characteristics may predispose the eyes to the development of a MH. The relationship between the RA trajectory and the presence of a MH should be remembered when the pathogenesis of a MH is considered.

## Supporting Information

S1 Fig Correlation of retinal artery (RA) trajectory and stages of macular hole (MH). Eight eyes were classified into stage 2, 35 eyes were stage 3 and 12 eyes were stage 4. The correlation analysis by Steel-Dwass showed significant correlation between the RA trajectory of stage 2 and that of stage 3 eyes, but not in others as follows. The results indicated that the RA trajectory was wider in MH of stage 3 than in that of stage 2.
(TIF)
