# Supplementary figures and images for: Wider Retinal Artery Trajectories in Eyes with Macular Hole Than in Fellow Eyes of Patients with Unilateral Idiopathic Macular Hole
Source: PLoS One. 2015 Apr 13;10(4):e0122876. doi: 10.1371/journal.pone.0122876 (PMC4395100; doi:10.1371/journal.pone.0122876)

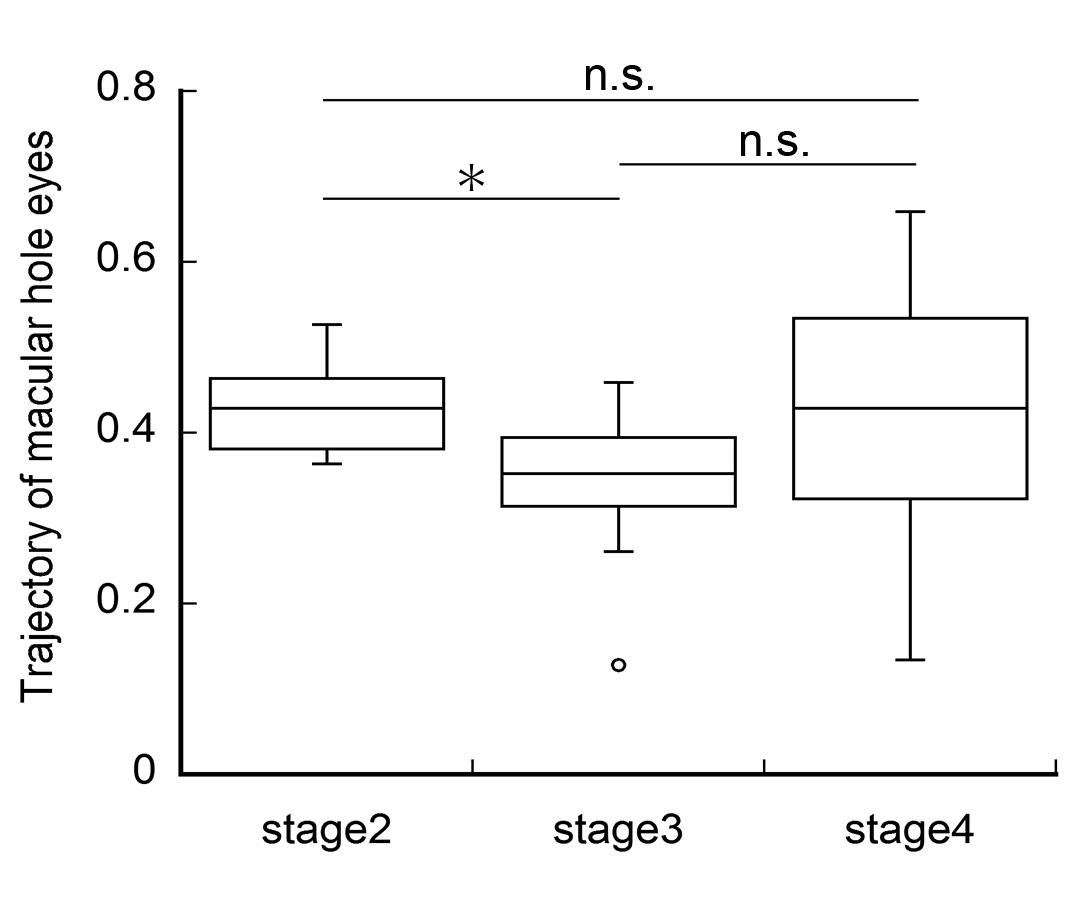

Supplement: S1 Fig — Eight eyes were classified into stage 2, 35 eyes were stage 3 and 12 eyes were stage 4. The correlation analysis by Steel-Dwass showed significant correlation between the RA trajectory of stage 2 and that of stage 3 eyes, but not in others as follows. The results indicated that the RA trajectory was wider in MH of stage 3 than in that of stage 2. (TIF) [file pone.0122876.s001.tif]
